# Anakinra in Heart Failure: A Systematic Review and Meta-Analysis of Randomized Controlled Trials

**DOI:** 10.3390/medsci11010004

**Published:** 2022-12-26

**Authors:** Kamran Mahfooz, Aditya Rana, Keerthi Palagati, Akshata Krishna Suvarna, Christian Perryman, Sai Pranathi Gaddipati, Arshiya Adhnon, Rupesh Andani, Advait Vasavada

**Affiliations:** 1 Department of Internal Medicine, Lincoln Medical Center, New York, NY 10451, USA; 2 Department of Internal Medicine, Armed Forces Medical College, Pune 411040, India; 3 Department of Internal Medicine, Government Medical College, Ananthapur 515001, India; 4 Department of Internal Medicine, Government Medical College, Miraj 416410, India; 5 Department of Internal Medicine, Saint James School of Medicine, Cane Hall Road, Arnos Vale VC0280, Saint Vincent and the Grenadines; 6 Department of Internal Medicine, Mallareddy Medical College for Women, Hyderabad 500055, India; 7 Department of Internal Medicine, Dubai Medical College, Al Muhaisanah 1, Al Mizhar, Dubai P.O. Box 20170, United Arab Emirates; 8 Department of Internal Medicine, MP Shah Medical College, Jamnagar 361008, India

**Keywords:** anakinra, heart failure, inflammation, ventilatory efficiency

## Abstract

*Background:* Heart failure (HF) has become increasingly difficult to manage given its increasing incidence. Despite the availability of novel treatment target relieving inhibition and congestions for neurohormonal activation, heart failure is one of leading health conditions associated with high hospitalization and readmission rates, resulting in poor quality of life. In light of this, this article serves to demonstrate the effect of anakinra as one of the treatment paradigms for HF to explore the need for advanced novel interventions. *Methods:* We conducted a search in five electronic databases, including *Embase*, *MEDLINE*, *Cochrane*, *Scopus*, and *PubMed*, for RCTs (randomized controlled trials) evaluating the effects of anakinra against placebo in HF. Meta-analysis was performed using RevMan version 5.4. *Results:* Eight RCTs were obtained and included for analysis in this study. The results demonstrate that anakinra significantly reduces the levels of CRP (C-reactive protein), with significant difference between anakinra- and placebo-treated groups. Analyses also show that CRP failed to cause an improvement in peak oxygen consumption and ventilatory efficiency. Additionally, the treatment-related adverse events were insignificant. Some considerable limitations are that the same set of researchers were involved in most of the studies; hence, more independent studies need to be encouraged. *Conclusion:* Anakinra was associated with a reduction in CRP levels, indicating some anti-inflammatory effects but no effect on function, exercise capacity, and adverse effects.

## 1. Introduction

Heart failure (HF) incidence and prevalence increases year by year, which has caused an increased pressure and strain on healthcare systems around the world. Prior to the initial reports on patients experiencing symptoms of HF despite having normal left-ventricular ejection fraction (LVEF) and small hearts, the term “heart failure” (HF) was only used to refer to patients with diminished ejection fraction [1,2,3]. Furthermore, due to its distinct appearance from “systolic heart failure”, this condition was initially referred to as “diastolic heart failure”. As a result, this resulted in discussions among scientific researchers as a community since there is a distinct difference between systolic and diastolic dysfunction that is more fictitious than real [4]. In regard, it was even demonstrated that patients with systolic function impairment may experience more severe diastolic dysfunction than those without, and patients with maintained ejection fraction can nonetheless experience systolic dysfunction [5,6]. Earlier, a classification based on ejection fraction was proposed, and the heart failure classification included HFrEF (heart failure with reduced ejection fraction) and HFpEF (heart failure with preserved ejection fraction). Presently, there is an emerging nomenclature where a distinct subset of HFmEF (heart failure with mid-range ejection fraction) has been identified [7]. This has led to the move towards a classification that recognizes a continuous spectrum of heart failure [8,9]. The absolute cutoffs vary across different guidelines, but the European Society of Cardiology’s most recent classification advocates the following: HFrEF; LVEF < 40%, HFpEF; LVEF ≥ 50%, HFmrEF; LVEF 40–49% [9].

Particularly, managing AHF (acute heart failure) is a difficult problem that necessitates early detection of the HF clinically, confirmation by diagnostic testing (including echocardiography, biological, and X-ray), evaluation of severity, and appropriate urgent therapy. AHF is highly difficult to manage in an emergency environment due to the variety of clinical presentations, which range from mild congestion with just the symptoms of being breathless (dyspnea) to a severe shock-state syndrome [10]. Some observational research studies have associated the biomarkers of the HF-related systemic inflammation to the effects of impaired cardiac function, which in turn leads to poor prognosis in patients suffering from the HFrEF condition as well as in those with chronic inflammatory infections [11,12,13,14]. 

**Figure 1:**
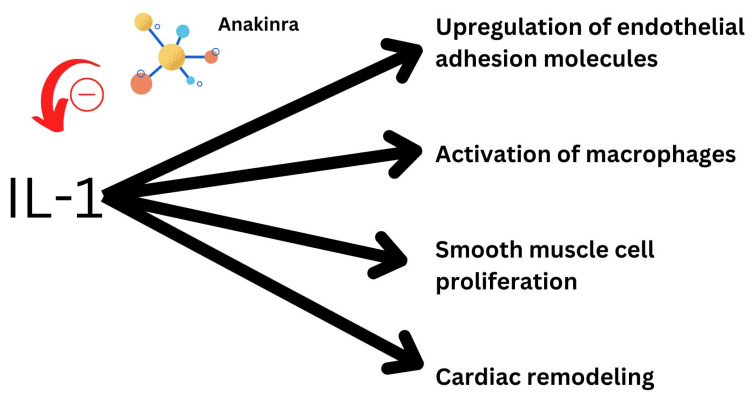
Effect of anakinra and its possible inhibitory effect on IL-1’s functions.

Exploring new biomarker-targeted treatments for heart failure have gained recent attention due to the recent success of canakinumab in the CANTOS trial [15]. Furthermore, since IL-1 plays a role in both systemic and localized inflammation, it is a potential target for anti-inflammatory therapy in heart failure [16]. Even a novel marker of isoform IL-1 receptor-like 1 has been found to be elevated in heart failure [17]. In experiments with animals, IL-1 has proven significant in altering systolic and diastolic function [18]. It is responsible for a myriad of inflammatory processes in the heart and hence is a potential target of treatment. Additionally, rheumatoid arthritis patients exhibit symptoms of decreased LV (left ventricular) diastolic function with therapy with anakinra, an IL-1 blocker, which proved to quickly restore normal LV diastolic function in these patients. Figure 1 demonstrates the possible effect of anakinra on cardiovascular functions of IL-1.

There is limited evidence on the efficacy of anakinra in improving outcomes of heart failure with reduced ejection fraction. Anakinra is an IL-1 receptor antagonist that has recently been demonstrated to increase aerobic exercise capacity in individuals with HFpEF, minimize the incidence of HF following ST-segment elevation AMI (acute myocardial infarction), and treat chronic systemic inflammatory disorders [19,20,21]. In regard to this, this article seeks to demonstrate the effects of anakinra in HF. To achieve this, we performed a systematic review and meta-analysis to evaluate how the drug impacts outcomes in heart failure, mainly focusing on biomarkers, ventilatory efficiency, peak oxygen consumption, and adverse events.

## 2. Materials and Methods

### 2.1. Study Design and Data Sources

The article herein is a systematic review and meta-analysis of randomized controlled trials (RCTs) conducted based on the guidelines and standards as dictated by the PRISMA framework [22]. Five medical databases were systematically searched for appropriate primary studies pertinent to the topic under study. To find RCTs exploring the use of anakinra in HFrEF that were available in English, searches were conducted in the following databases: *Embase*, *MEDLINE*, *Cochrane*, *Scopus*, and *PubMed*.

### 2.2. Search Strategy

A thorough search strategy focusing on keywords and essential concepts pertinent to this article was used. Additionally, the search method included the Boolean expression, which mostly consists of “AND” and “OR”. For effectiveness, the following set of keywords were utilized: “Anakinra” AND “Heart Failure” OR “HF” OR “Acute Heart Failure” OR “AHF” OR “Heart failure with reduced ejection fraction” OR “HFrEF” OR “HF with preserved ejection fraction” OR “HFpEF”. The search was limited to published random controlled trials in the English language.

### 2.3. Eligibility Criteria

The following inclusion criteria were adopted for careful selection of primary articles to be analyzed in this study:

- Primary research articles evaluating the effects of anakinra in HF;
- Original articles comprising RCTs only;
- Articles published in English and dating from 2010 to 2022.

In contrast, studies were excluded based on the following criteria:

- Secondary sources of anakinra effects on HF, including newspapers, magazines, and other systematic reviews;
- Studies evaluating the use of anakinra other than in HF;
- Primary articles published in languages other than the preferred English language to avoid the loss of information and distortion during translations;
- Case reports and other study designs.

### 2.4. Data Extraction Quality Assessment

We tasked two independent investigators to conduct data selection and extraction, where information was obtained from the studies that met the inclusion criteria based on PICO framework [23]. The data obtained by these reviewers include information on authors, study protocol, patients’ characteristics (participants), anakinra dosage (intervention), placebo treatment (comparator), and key findings (outcomes). An additional reviewer was consulted to help resolve issues resulting from data extraction to harmonize and elucidate meaningful information for data and statical analysis. In addition, quality appraisal was performed using the Cochrane risk of bias tool; the six-criteria tool—reporting, blinding, selection, binding, attrition, and other biases—is used to categorize studies as having a low, high, or unknown risk of bias [24]. 

### 2.5. Statistical Analysis

Based on the requirements and the data obtained, the Cochrane Review Manager Software (RevMan version 5.4) was effectively put to use for data analysis. In order to perform meta-analysis, the Cochrane guidelines dictate that data must be in the same format and measure similar aspects. Regarding this, most of the data recorded in the included articles was presented numerically as median and interquartile range (IQR). As a result, to use RevMan to perform the meta-analysis, imputations were made for median as mean and IQR/4 as standard deviation to facilitate similar format of numerical data for the analysis. Due to approximations and imputations, mean difference (MD) and poled odds ratio were selected as the effect measure at a 95% level of confidence. Therefore, statistical significance was evaluated at *p* < 0.05, while the I-square test was employed to test for heterogeneity, where above 50% (I^2^ > 50%) was deemed significant heterogeneity, and low heterogeneity was determined otherwise [25]. 

## 3. Results

### 3.1. Search Results

**Figure 2:**
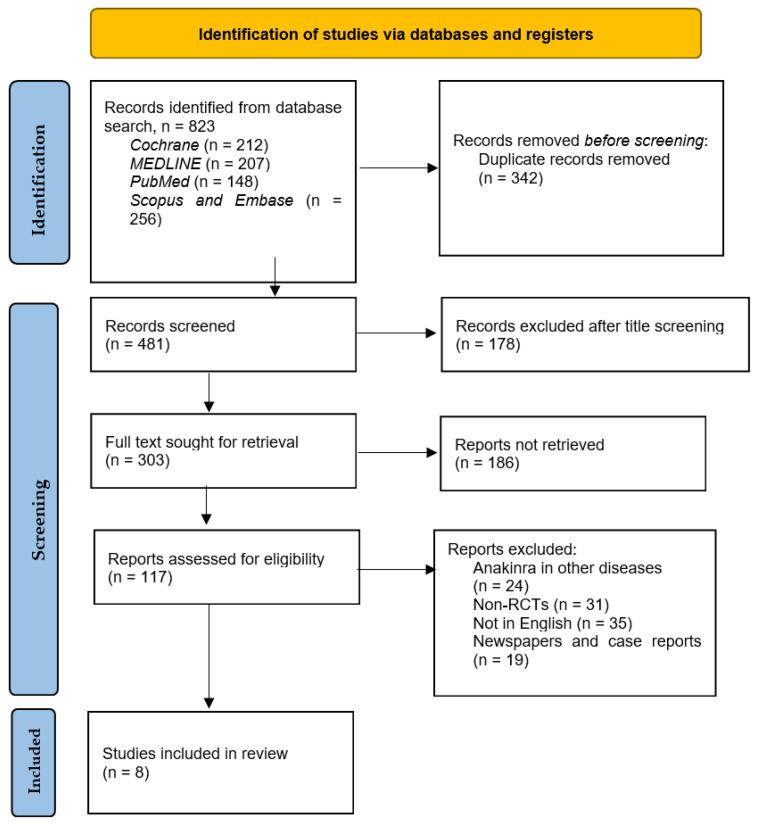
PRISMA flowchart depicting the search process utilized to arrive at the eight RCTs.

The total citations identified through the various databases in addition to reviewing their reference lists included 823 articles. Of these, only eight studies were RCTs meeting the inclusion criteria, namely exploring the effect of anakinra on HFrEF, and were acquired for analysis. Duplicates were 342 in number, and 178 were excluded after title and abstract screening, and 186 studies were not retrieved due to the requirement of purchase and special licenses needed for access and also due to URL errors during searching for their full texts. Figure 2 below depicts the search process used to select the eight pertinent articles for this study. Table 1 depicts the outcomes of the use of anakinra in HF that were evaluated in various trials and those included in this systematic review. 

Table 2 describes the individual study characteristics and the outcomes that were studies in each of those studies related to anakinra.

### 3.2. Risk of Bias Evaluation

**Figure 3:**
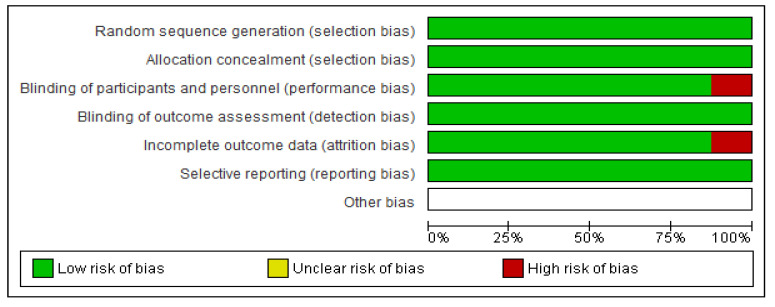
Depicting the risk of bias graph judgments for the six items.

**Figure 4:**
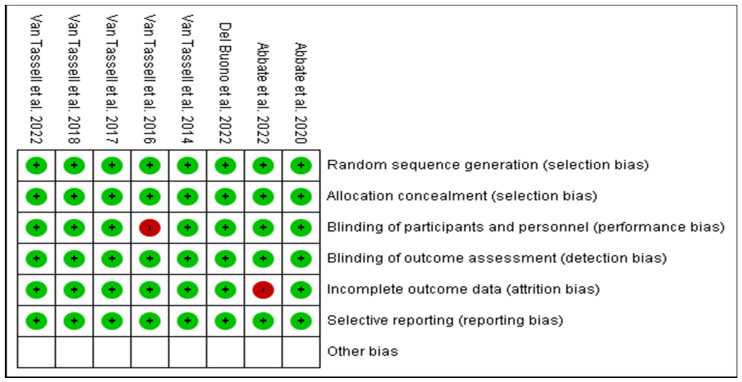
Risk of bias summary of the eight RCTs against various item evaluations [19,26,27,28,29,30,31,32].

Based on the protocols adopted by the Cochrane risk of bias tool of evaluation, all the eight RCTs were majorly of low bias across all the items as depicted in Figure 3 and Figure 4. The low risk was manifested by the green shading, and high risk corresponds to red, while yellow represents uncertain bias or unclear result. This was achieved by disregarding primary articles characterized by high risk of bias to avoid altering and inconveniencing the results of this study. The same protocol was applied for those with unclear bias to eliminate doubtful outcomes of our study.

### 3.3. Effect of Anakinra on CRP Levels

**Figure 5:**
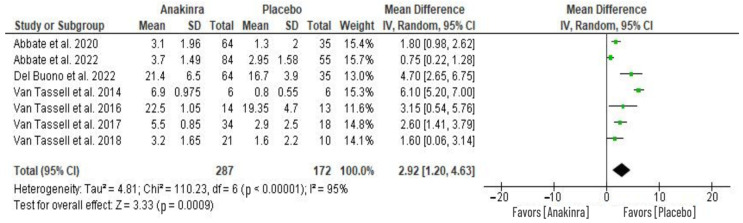
Anakinra effect on CRP levels [19,26,27,28,29,30,31,32].

Seven RCTs evaluated the impact on C-reactive protein (CRP) levels caused by anakinra treatment. The changes in the level of CRP from baseline were used to perform a meta-analysis of the seven RCTs as depicted in Figure 5. This analysis reveals a significant difference in CRP levels between the two groups, with anakinra treatment showing a significantly higher reduction from the baseline CRP level compared to placebo treatment (MD 2.17, 95% CI 1.81 to 2.53; I^2^ = 95%) at *p* = 0.0009. The overall heterogeneity across the seven studies was significantly high, with I^2^ = 95%.

### 3.4. Effect of Anakinra on VO_2_

**Figure 6:**
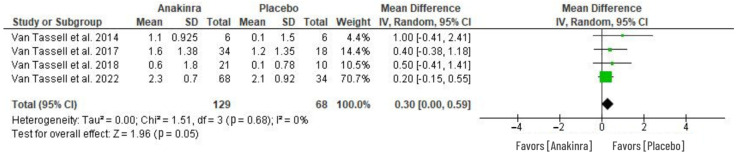
Effect of anakinra on peak oxygen consumption (VO_2_) changes from baseline values [28,30,31,32].

The analysis of the anakinra effect on peak oxygen consumption (VO_2_) in patients with HF was determined using four RCTs as in Figure 6. In comparison to placebo or standard treatment, anakinra was associated with greater change in peak VO_2_ from baseline VO_2_. However, the difference between anakinra and placebo in peak VO_2_ changes was not statistically significant (MD 0.30, 95% CI 0.00 to 0.59; I^2^ = 0%), with an overall test effect of Z = 0.96 at *p* = 0.05. The heterogeneity was insignificant (I^2^ = 0%).

### 3.5. Effect of Anakinra on VE/VCO_2_ Slope

**Figure 7:**
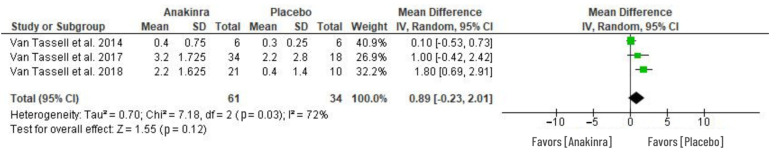
Effect of anakinra on ventilatory efficiency [28,30,31].

The effect of anakinra on ventilatory efficiency in HF patients was determined by the VE/VCO_2_ slope as in Figure 7. Among the three RCTs that evaluated VE/VCO_2_ slope, no difference was depicted between anakinra treatment relative to placebo, and thus, the statistical difference was insignificant between the two groups (MD 0.89, 95% CI –0.23 to 2.01; I^2^ = 72%) at *p* = 0.12.

### 3.6. Adverse Events (AEs)

**Figure 8:**
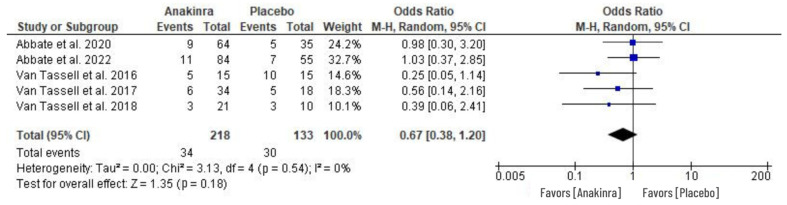
Adverse events associated with the use of anakinra vs. placebo treatment in HF patients [26,27,29,30,31].

An odds ratio effect measure was performed in five RCTs to evaluate the adverse events associated with the use of anakinra in HF treatment. The AEs evaluated includes the cases of nonserious infections, respiratory infections, cardiac and non-cardiac evets, and the occurrence of other clinical events. A random effect model of the analysis revealed insignificant difference between anakinra and placebo treatment although the individual studies indicated that there was considerably small number of patients with adverse events in the anakinra group compared to placebo (OR 0.67, 95% CI 0.38 to 1.20; I^2^ = 0%) at *p* = 0.18, while the heterogeneity was insignificant. See Figure 8 for the results of our analysis.

## 4. Discussion

Our study was conducted with the aim of providing insight on whether anakinra is a useful therapeutic intervention for HF that will be instrumental in alleviating associated symptoms and, most importantly, improve the patients’ cardiorespiratory functioning. The results of our analysis revealed that anakinra has significant effect on the CRP levels but minimal to no impact on ventilatory capacity, exercise capacity, and adverse events. Many of the studies evaluated the effect of anakinra administered once or twice a day through 100 mg subcutaneous injections for 2 to 24 weeks. However, two weeks of treatment were considered in our study for uniformity in the statistical analysis since most trials were evaluated during this period. This indicates that substantial evidence on the effect of the drug in longer duration of treatment is sparse. More granular analysis of the adverse event data could not be performed, as there were inconsistencies in the representation of individual adverse events across the studies, which could not be harmonized and used to provide meaningful statistical analysis. Van Tassell et al. (2022) is an ongoing trial that is measuring CRP as an outcome, but the current trial data only provided a theoretical description of the biomarker levels and could not provide numerical data (in terms of mean +/− SD) that could be used in our analysis, similar to other studies. Hence, it could not be included for analyzing the effect of anakinra on CRP [32].

Our study evaluated the effect of anakinra (IL-1 blocker), which is a significant recombinant of IL-1 receptor antagonist, administered once or twice a day through 100 mg subcutaneous injections for periods ranging from 2 to 24 weeks. Based on the results of our study, anakinra treatment bolstered a significant effect in the reduction of CRP levels relative to standard treatment (placebo). Since CRP production is directly related to IL-6 rather than IL-1, anakinra’s effect on the marker is indirect through a possible mechanism that alters persistent myocardial inflammation in HF [15]. This result implies that anakinra is significant as an inhibitor to the systemic inflammatory response, indicated by the statistically significant difference between patients treated with anakinra versus those in the placebo group. This finding seems coherent with the results of randomized placebo phase II trials, which observed that anakinra was associated with significant reduction of CRP from baseline levels than placebo at 12 weeks of treatment [28,30,31]. However, our findings diverge from previous research that employed alternative cytokines, including infliximab, in such a way that the levels of CRP only declined in 2 weeks of treatment although afterwards, it gradually returned back to the baseline values irrespective of changes, and treatment with infliximab progressed [33]. Similarly, etanercept could not cause significant changes to CRP levels despite having modest impact on IL-6 [34]. 

Despite its prowess as an inflammatory response inhibitor [27,29], anakinra, however, did not show a significant difference in peak oxygen consumption. In comparison to placebo treatment, anakinra failed to significantly improve the aerobic capacity as indicated by the associated modest effect on VO_2_ (see Figure 6). Similarly, ventilatory efficiency was not improved from the baseline values, with an insignificant difference between anakinra and placebo treatment (Figure 7). These analyses are consistent with the results of three RCTs that indicated that the ventilatory efficiency and peak oxygen consumption levels remained unchanged from baseline after 12 weeks of anakinra intervention [24,25,29]. However, this outcome contradicts the results of a D-HART study using anakinra as a robust impact on exercise capacity in patients with HF, revealing improved peak VO_2_ in 2 weeks of treatment [28]. Nonetheless, the observed improvement was relatively smaller in patients with HFrEF [21,28]. Despite the modest change due to anakinra, the peak VO_2_ was considered significant in their results. On the contrary, in a REDHART trail, anakinra treatment for 2 weeks had no significant difference but, with continued treatment at 4 and 12 weeks, was associated with an improved peak VO_2_ relative to the baseline values [30,32]. 

Furthermore, this study evaluated adverse events as treatment-related side effects of anakinra. Among the AEs were injection-site reactions [27], serious and non-serious sinus and respiratory infections [31], events of ischemic or hemorrhagic stroke [30], and worsened pulmonary congestion [26]. The high rate of readmissions and longer hospitalizations can be articulated along with these adverse events, which may result in deaths among HF patients if not well-managed [27]. Our study indicates that there was a smaller number of patients with adverse events in the anakinra-treated group than in the placebo group. However, the statistical difference between the two was insignificant (Figure 8). Generally, there were several cases in both anakinra and placebo groups within the included studies with high rates of hospital readmissions following AEs associated with HF across all the populations under considerations [30]. This correlates with one study that showed no infections in the anakinra group compared to 12 AEs among ten patients in the placebo group [29]. Furthermore, despite the occurrence of adverse events, data collected from the Duke Activity Status Index (DASI) and Minnesota Living with Heart Failure Questionnaire (MLWHFQ questionnaires) indicated a positive change in functional capacity restoration in the anakinra group compared to the placebo group [31,32]. Similarly, a significant improvement in DASI was also recorded in the anakinra group [28,30]. 

The existing interventional treatment paradigm for acute HF, including HFrEF and HFpEF, work by targeting to relieve the patients from acute decompensation and cardiac remodeling. Recent insights from the use of anti-inflammatory drugs such as canakinumab produced improvement in heart damage associated with the persistent inflammation seen in some types of heart failure. Furthermore, novel biomarkers related to the development and progression of heart failure are being studied. The cardio-inflammatory phenotype of heart failure is of particular interest since markers such as CRP, a soluble isoform of IL-1 receptor-like 1 (also known as sST2), have been seen to be increased in the phenotype, indicating a vital role in the disease process. Even in preclinical studies, TNF, IL-1β, IL-6, and IL-18 have been found to be increased in HF [35]. HFpEF has been difficult to study due to its heterogenous nature and lack of reliable pre-clinical models [36]. No study was particularly aimed at the cardio-inflammatory phenotype, and only one biomarker was repeatedly used in the studies (i.e., CRP). sST2 has been found to be useful as a prognostic marker with a better predictive value of fatal outcomes [37]. In the future, in more focused studies with broad range of biomarkers, HFpEF should be evaluated to better understand the effect of drugs such as anakinra on the inflammation associated with HF [38].

### Limitations to the Study

The article is limited due to constrictions in using RCTs only in addition to insufficient literature exploring anakinra treatment for HF. The statistical analysis was conducted largely based on imputations since the depicted values in the studies were provided in median and IQR, which might be a source of errors and inaccuracies in the analysis. Moreover, there is the chance of unreliable results due to the varying small populations utilized in individual studies in addition to different treatment periods and frequency per day of anakinra injection from one study to another. All of RCTs included belong to the same group of researchers with similar procedures, locations, and patients’ characteristics. Therefore, this is also a source of bias. The time of treatment of anakinra ranged from 2 to 24 weeks, but our analysis only took 2-week treatment into consideration for uniformity in the data obtained. Hence, it is vital that future studies take into consideration the relationship of time with the outcomes of anakinra in heart failure if used for longer durations.

## 5. Conclusions

Our study sought to explore the effects of anakinra in heart failure as a means of providing insight on whether it can prove to be a novel treatment approach to tackling HF. Based on our analysis, anakinra, as a combinatory IL-1 blocker, was instrumental in the reduction of CRP levels among the HF patients. Consequently, this demonstrates that anakinra is a key inhibitor to inflammatory response, which is crucial in improving functional capacity in HF patients. Future trials studying the drug specifically in patients with a cardio-inflammatory phenotype maybe fruitful in analyzing its impact. Nevertheless, short-term treatment with anakinra was not effective in improving the ventilatory efficiency or changing the peak oxygen consumption in heart failure.

## Figures and Tables

**Table 1:** Outcomes studied in the trials of use of anakinra in heart failure.

Treatment	Ejection Fraction Effects	Effects
Anakinra	CPX parameters and HF events	Peak aerobic capacity (VO_2_) and ventilatory efficiency (VE/VCO_2_ slope).
	Adverse events	Clinical, cardiac, and non-cardiac deaths, hospitalization, and injection-site reactions.
	QoL measures	General improvement in quality of life
	Biomarkers	High-sensitive C-reactive protein and NT-proBNP levels

**Table 2:** Study characteristics. NOTE: RCT, randomized controlled trial; CRP-C, reactive protein; Vo2, peak oxygen consumption; VE/Vco2, minute ventilation–carbon dioxide production slope; NT, proBNP-N-terminal pro-B-type natriuretic peptide; AEs, adverse events; QoL, quality of life; CPX, cardiopulmonary exercise testing.

Study ID		Participants			Anakinra (Once/Twice Daily Subcutaneous Injections) (mg)	Treatment Period (weeks)	Placebo (N)	Main Outcome Anakinra Effects
Author, year	Design	N	M/F	Age (years)				
Abbate et al. (2020) [26]	RCT	99	80/19	45–65	100	2	35	AEs, CRP, stroke volume, and stroke work.
Abbate et al. (2022) [27]	RCT	139	110/29	48–61	100	2	55	CRP and AEs
Del Buono et al. (2022) [19]	RCT	99	80/19	48–65	100	2	35	CRP, leukocyte differential count
Van Tassell et al. (2014) [28]	RCT	12	1/11	62	100	4	6	CRP, VO_2_, VE/VCO_2_ slope, and exercise
Van Tassell et al. (2016) [29]	RCT	30	21/9	49–66	100	2	15	CRP, AEs, and NTproBNP
Van Tassell et al. (2017) [30]	RCT	60	38/22	49–68	100	12	18	AEs, QoL, CRP, NT-proBNP levels, VE/Vco_2_ slope, and peak Vo_2_.
Van Tassell et al. (2018) [31]	RCT	31	13/18	50–60	100	12	10	CRP levels, AEs, NT-proBNP levels, peak Vo_2_ or the VE/Vco_2_ slope, and exercise.
Van Tassell et al. (2022) [32]	RCT	102	_	≥21	100	24	34	CRP, CPX, peak VO_2_, and QoL

## Data Availability

Trials described in the study were publicly available.
